# Supplementary material for: Atomically resolved imaging of the conformations and adsorption geometries of individual β-cyclodextrins with non-contact AFM
Source: Nat Commun. 2024 Nov 2;15:9482. doi: 10.1038/s41467-024-53555-0 (PMC11531514; doi:10.1038/s41467-024-53555-0)
Supplement: Supplementary file 1 — Supplementary Information [file 41467_2024_53555_MOESM1_ESM.pdf]

Supplementary Information

Atomically resolved imaging of the conformations and adsorption geometries of individual β-cyclodextrins with non-contact AFM

Márkó Grabarics[1,2,¥], Benjamín Mallada[3,4,¥], Shayan Edalatmanesh[3,4,¥], Alejandro Jiménez-Martín[3,4,5], Martin Pykal[4], Martin Ondráček[3], Petra Kührová[4], Weston B. Struwe[2,6], Pavel Banáš[4]*, Stephan Rauschenbach[1,2]*, Pavel Jelínek[3,4]* & Bruno de la Torre[4,7]*

[¥]These authors contributed equally: M.G., B.M., and Sh.E.

[1]Department of Chemistry, University of Oxford, OX1 3QU Oxford, UK

[2]Kavli Institute for Nanoscience Discovery, University of Oxford, OX1 3QU Oxford, UK

[3]Institute of Physics, Czech Academy of Sciences, 16200 Prague, Czech Republic

[4]Czech Advanced Technology and Research Institute, Palacký University Olomouc, 78371 Olomouc, Czech Republic

[5]Faculty of Nuclear Sciences and Physical Engineering, Czech Technical University in Prague, 115 19 Prague, Czech Republic

[6]Department of Biochemistry, University of Oxford, OX1 3QU Oxford, UK

[7]Nanomaterials and Nanotechnology Research Center, CSIC-UNIOVI-PA, 33940 El Entrego, Spain

*Corresponding authors: pavel.banas@upol.cz; stephan.rauschenbach@chem.ox.ac.uk; jelinekp@fzu.cz; bruno.de@upol.cz

Supplementary Note 1. STM topographs of β-cyclodextrin (β-CD) islands on Au(111)

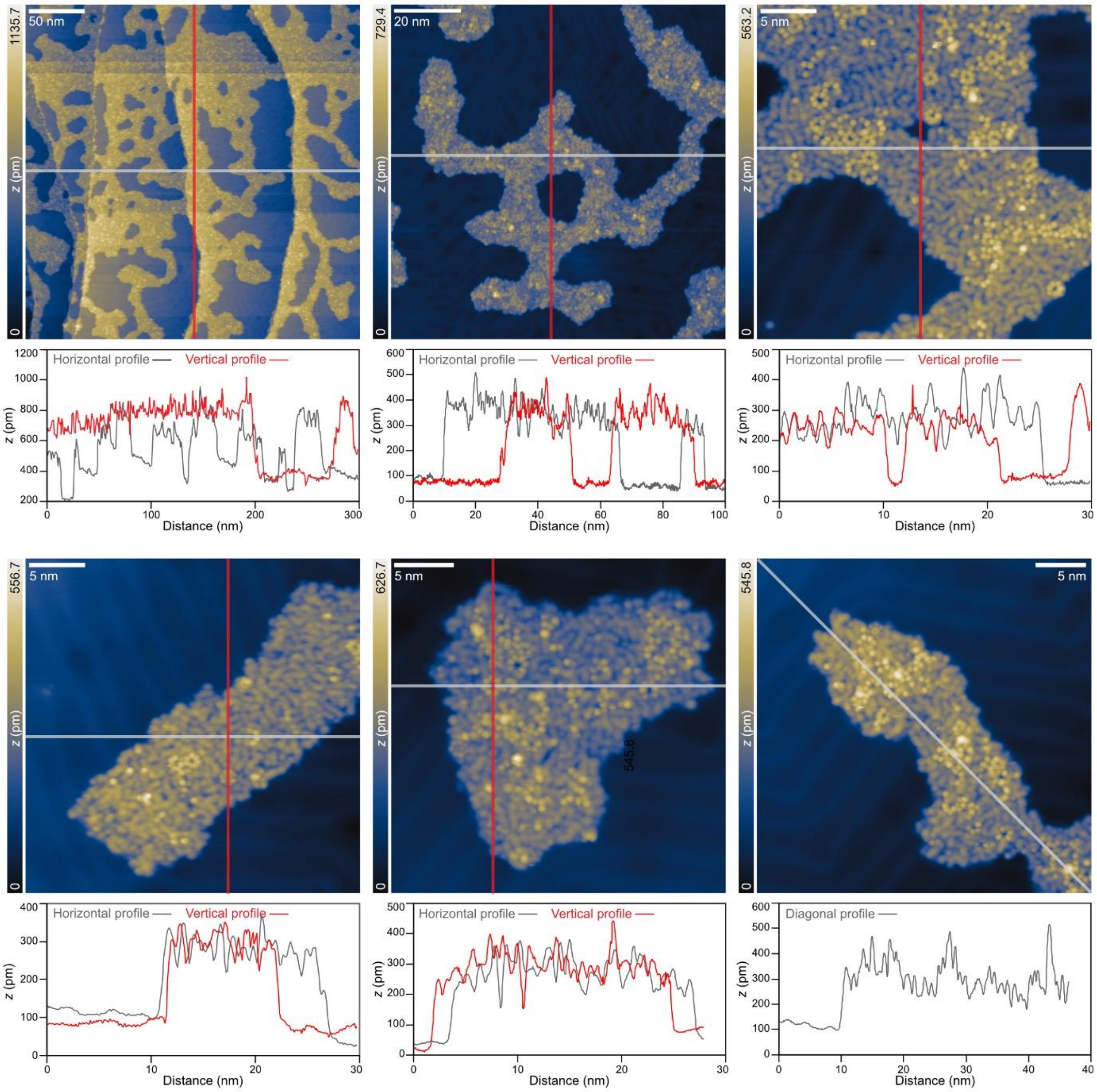

Supplementary Fig. 1 | STM topographs of β-CD islands formed on the Au(111) surface upon electrospray deposition. Corresponding line profiles as indicated are shown below each STM image. The average topographic height of the islands is around 250 to 350 pm.

Supplementary Note 2. STM and nc-AFM images of the bed surrounding intact β-cyclodextrins on Au(111)

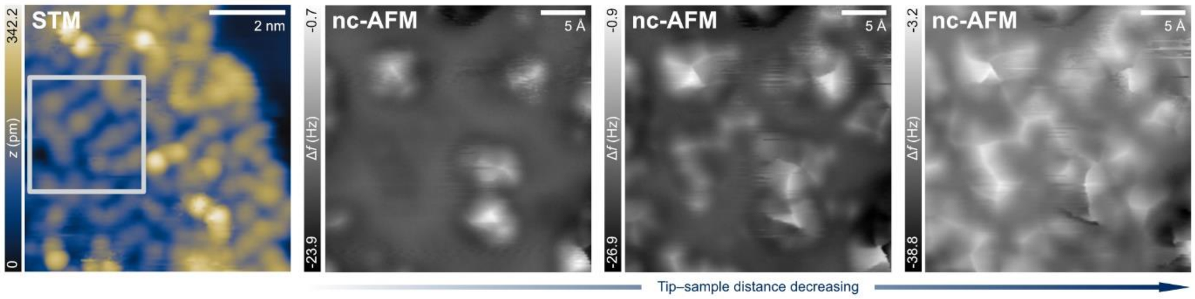

Supplementary Fig. 2 | STM and high-resolution nc-AFM images showing the bed surrounding β-CD molecules in the islands. The grey square in the STM topograph indicates the frame of the nc-AFM images; nc-AFM images were recorded at different tip-sample distances (decreasing from left to right).

Supplementary Note 3. Measurement of the adsorption height of β-cyclodextrin on Au(111) by nc-AFM

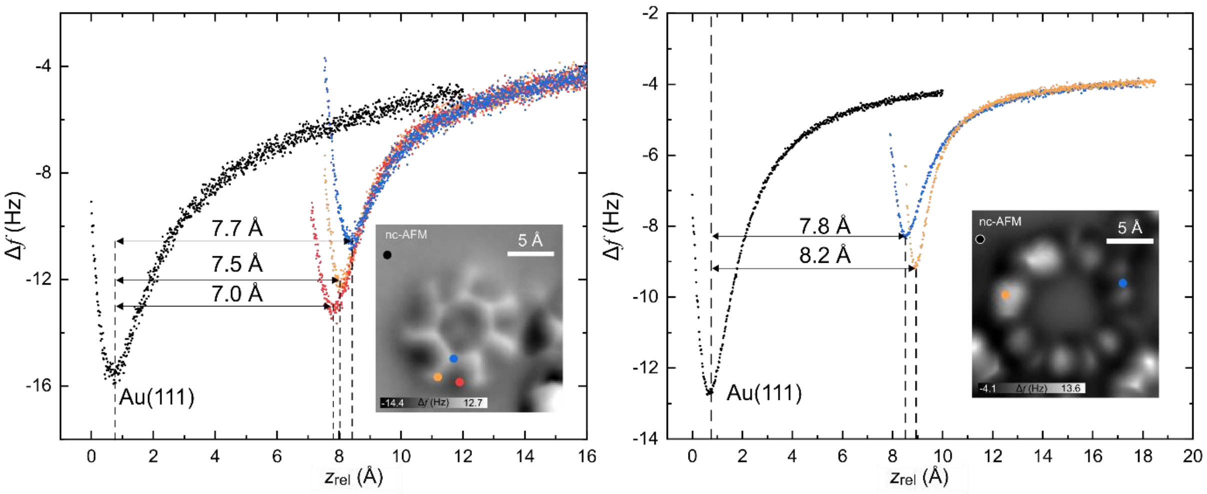

Supplementary Fig. 3 | Measurement of the frequency shift (Δ*f*) as a function of the tip-sample distance (*z*) to determine the adsorption height of β-CD on Au(111) in its different adsorption geometries. The black curves show Δf as a function of z, measured above the clean Au(111) substrate near the depicted molecules. The coloured curves show $\Delta f(z)$ at the respective xy-positions above the molecules as highlighted with coloured dots in the nc-AFM images. The height of an object (measured at a defined point) with respect to the Au(111) surface can be calculated as the difference between the two z_{min} values[1]. The adsorption height of the molecule on the left panel (measured as 7.5 and 7.7 Å) and on the right panel (7.8 and 8.2 Å) are very similar as determined from the minima of the $\Delta f(z)$ curves. The 7.0 Å value corresponds to a lower-lying part of the molecule on the left and thus does not represent the total adsorption height of this species (it falls within an attractive region between more repulsive rays in the nc-AFM image). The similarity of adsorption heights further supports the interpretation that the above two structures represent two different adsorption geometries of the same compound, β-CD.

Supplementary Note 4. Molecular dynamics (MD) simulations of β-cyclodextrin

4.1. Computational details

MD simulations were performed using the Gromacs 5.1.4 software package[2] with the Plumed extension.[3] β-CD was modelled with the GLYCAM04 force field.[4, 5, 6, 7] The Au(111) surface, approximately 10 × 10 nm in size, was represented using GolP force field, which explicitly accounts for induced polarization.[8] The Au(111) atoms were fixed in place, except for the rigid rod model of dipoles. The structure of β-CD was obtained from the Protein Data Bank (PDB ID: 3CGT). The β-CD molecule was positioned on Au(111) surface with either its primary face up (p↑) or secondary face up (s↑). To enhanced hydrogen bond stability on the primary face, a 1 kcal/mol potential (referred to as HBfix) was applied between the heavy oxygen atoms.[9] Non-bonded interactions were truncated at 1 nm. Electrostatic interactions were treated using the Particle-Mesh Ewald method (PME) with the 1 nm cutoff for the real space term. The LINCS algorithm was used to constrain hydrogen atoms.[10] The equations of motion were integrated with a 2 fs time step. Each system was minimized, followed by a 5 ns NpT (in the case of solvent simulation) thermalization using a V-rescale thermostat[11] with a gradual temperature rise from 10 to 300 K and a coupling constant of 0.1 ps, and an isotropic Berendsen barostat[12] with a reference pressure of 1 bar and a coupling constant of 1.0 ps. The final production run was performed in the NVT ensemble for 1.1 μs (or for 100 ns for a set of 10 β-CDs and simulations with HBfix). Finally, the equilibrated molecules were cooled to 5 K in 2 ns to obtain low-energy gas-phase β-CD conformers for further ab initio simulations.

4.2. Results from MD simulations

First, the conformational behaviour of β-CD in a water/methanol mixture was studied. It was observed that β-CD predominantly maintained its conformation, characterized by the glucose rings arranged in a circular fashion parallel to the central symmetry axis of β-CD. Only rarely, a pair of adjacent glucose units flipped to the perpendicular orientation in relation to the central symmetry axis, however, this conformation was not stable in solution for long periods of time. The secondary OH groups (on the secondary face) can be found in two dominant states, with the hydrogens pointing either in clockwise or anti-clockwise direction (as seen from the secondary face) to the neighbouring secondary OH groups, forming a hydrogen-bonded chain circle. However, the predominant orientation of the secondary face was anti-clockwise – 16.5 % of the population was in the state where the secondary OH groups were rotated anti-clockwise. This population also included states where one or two secondary OH groups (out of 14 groups on the secondary face) break the perfect anti-clockwise chain directionality and thus slightly disturbed the symmetry of the molecule. In contrast, the primary face of β-CD showed greater flexibility, with no dominant conformation.

The structure of the adsorbed β-CD was highly dependent on the orientation of the β-CD cycle with respect to the Au(111) surface. When the β-CD was placed with its secondary side towards Au(111), the distinct cyclic arrangement of the β-CD became more stable and exhibited less flexibility compared to the solvent environment. However, it retained some mobility on the surface. Similarly to the solvent simulation, the secondary OH groups predominantly adopted an anti-clockwise arrangement, evident in approximately 99% of the population. Common patterns were also found on the primary side of the

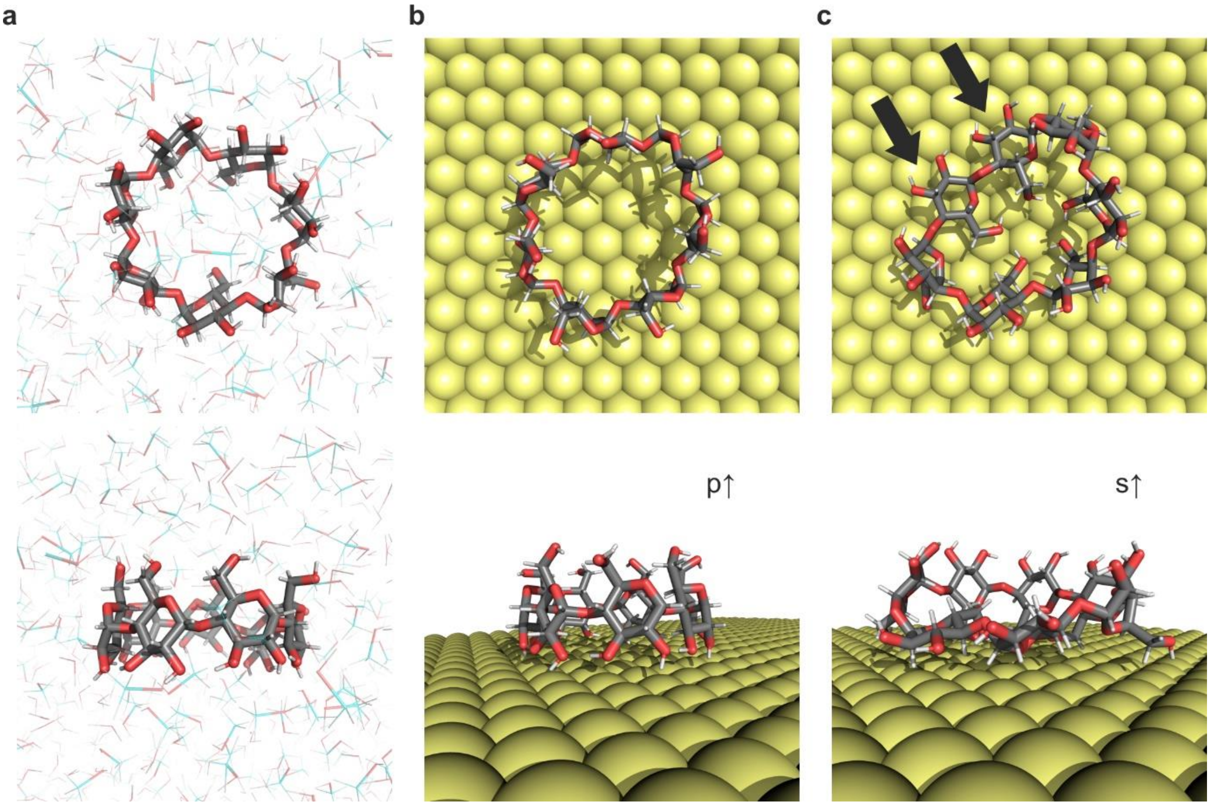

Supplementary Fig. 4 | MD simulations. Snapshots from MD simulations showing structural motives of β-CD molecules **a,** in water/methanol and adsorbed on the Au(111) surface with its **b,** primary face up, p↑ or **c,** secondary face up, s↑, with two adjacent glucose units stacked to the surface. The black arrows show the stacked glucose units.

β-CD. In more than 25% of the population the primary OH groups alternated between facing in and out of the cycle. In contrast, when the β-CD was placed on the surface with its primary face, significant deformation of the macrocycle occurred. This caused the structure to collapse, resulting in an oval shape. Two neighbouring glucose units stacked on the surface, breaking the original tight H-bonds with the remaining units. This disruption also affected the organization of the primary OH groups on the primary side of the β-CD, which no longer showed any preferential arrangement. In the next step, an additional potential, called HBfix, was added to the hydrogen bonds on the primary side of the β-CD. This potential was designed to increase the stability of the hydrogen bond pairs by providing a favourable energy contribution of 1 kcal mol^{-1} in favour of the bound state. It resulted in a dominant highly symmetric arrangement, characterized by anti-clockwise (as viewed from the primary side) orientations of the hydrogen-bonded primary OH groups pointing inwards within the cyclic structure. However, it proved ineffective in preventing deformation of the cyclic structure when β-CD is oriented with the primary side facing the surface. This resulted in a structural outcome identical to that observed in the previous scenario of two stacked units.

In general, β-CD produced two easily identifiable motifs on the surface, depending on the mutual orientation of the β-CD and the Au(111) surface, i.e., the highly symmetric β-CD structure bound to the surface via secondary face, while oval shape structure with broken symmetry and flip of two adjacent glucose units in case of binding via primary β-CD face. This was further supported by simulations with a collection of 10 β-CD molecules, when they maintained their specific adsorption geometry even in larger clusters.

Supplementary Note 5. DFT calculations of β-cyclodextrin and simulation of nc-AFM images

5.1. Computational details

DFT calculations for all free-standing molecules were performed using the FHI-aims package[13]. All geometry optimizations were carried out in the gas phase, using the generalized gradient approximation Perdew–Burke–Ernzerhof exchange–correlation functional[14]. In all the calculations, the light settings for the numerical atomic basis sets were employed. For all structures, the Brillouin zone was sampled using the Gamma-point. Systems were allowed to relax until the Hellmann-Feynman forces reached values below 10^{-2} eV $Å^{-1}$. Theoretical nc-AFM maps were calculated by the DFT and the Probe-Particle package[15] for a CO-like tip.

5.2. Atomic structure and the corresponding DFT-calculated simulated nc-AFM image of a distorted β-cyclodextrin conformer

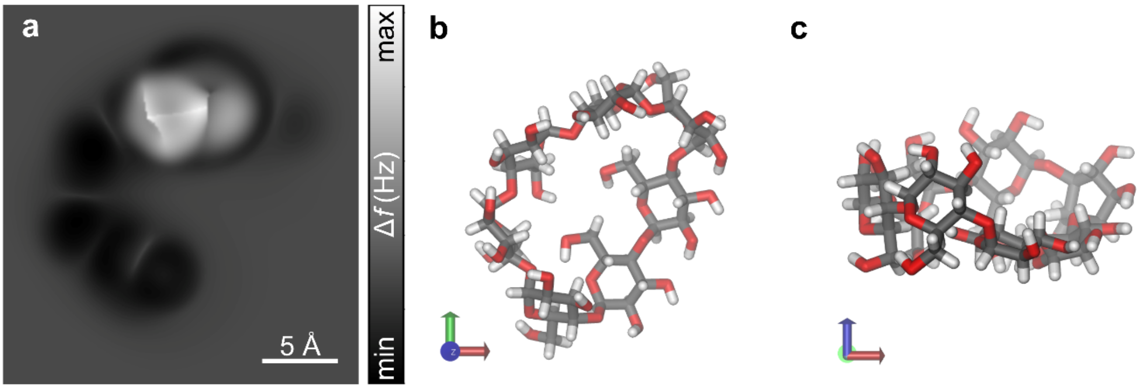

Supplementary Fig. 5 | A distorted β-CD conformer, calculated on the DFT level of theory. a, The nc-AFM simulated image; **b,** top view and **c,** side view of the atomic structure of the distorted molecule.

5.3. Atomic structures and the corresponding DFT-calculated simulated nc-AFM images of alternative symmetric β-cyclodextrin conformers

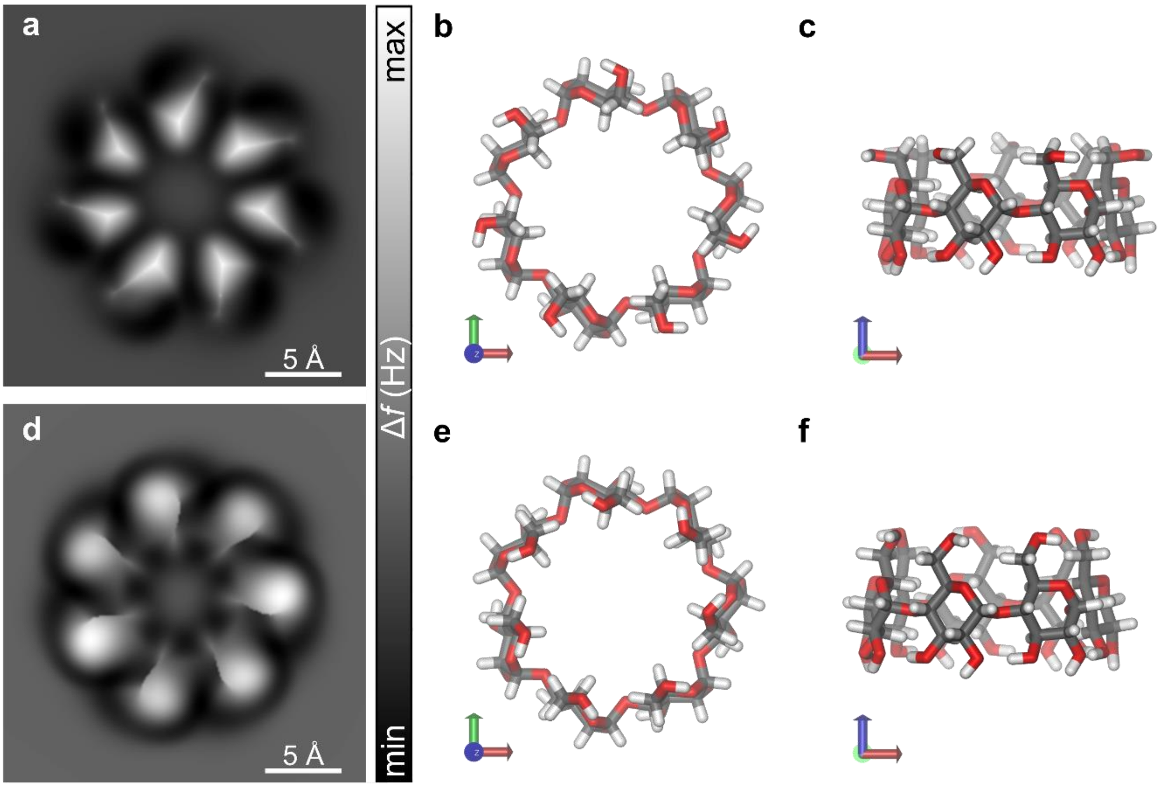

Supplementary Fig. 6 | Alternative conformations of the β-CD molecule, calculated using MD. (Top row) **a,** Simulated nc-AFM image (showing the primary face of the molecule) of one alternative β-CD conformer, with **b,** the top view and **c,** side view of the corresponding atomic structure. In this structure, the C-H bonds stick out of the *xy*-plane. (Bottom row) **d,** Simulated nc-AFM image of a second alternative β-CD conformer; **e,** top view and **f,** side view of the respective atomic structure. In this case, the O-H bonds are located higher along the *z*-axis than the C-H bonds, but they remain in the *xy*-plane.

5.4. DFT-calculated nc-AFM simulations and the electrostatic potential of β-cyclodextrin with the primary face upwards (p↑)

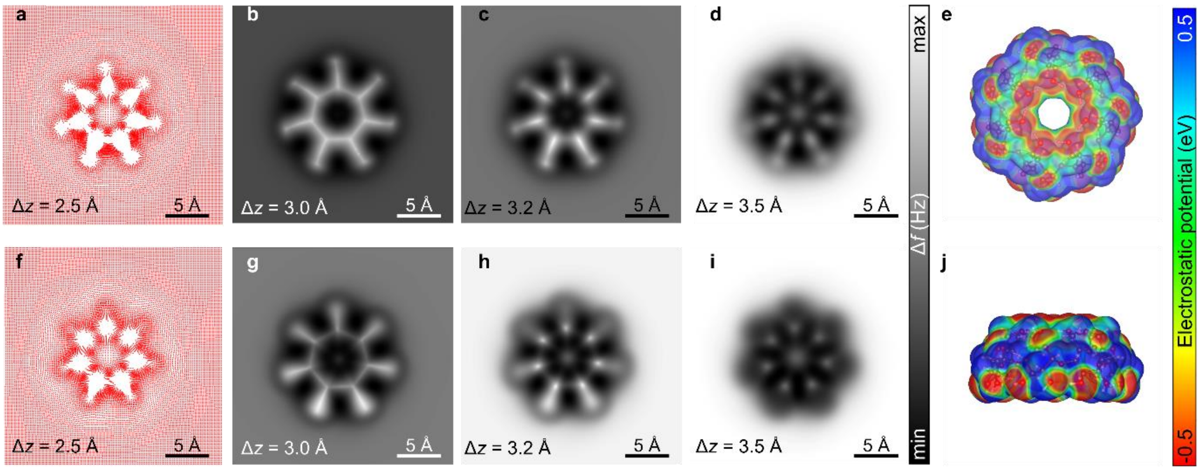

Supplementary Fig. 7 | nc-AFM simulations of β-CD with its primary face upwards at different heights. The lateral relaxations of CO-like probe particle at tip-sample distances corresponding to the closest approach of the probe particle during the oscillation for simulated nc-AFM images **b** and **g** are presented as red dots in panels **a** and **f**, respectively. Panels **b–d** hold the nc-AFM simulations without including the electrostatic potential, and sections **g–i** contain the simulations that include the electrostatic interaction between probe and sample. Panels **e** and **j** include the top (viewed from the primary face) and side views of β-CD's electrostatic potential map, respectively. The Δ*z* values define the distance between the outermost atom of the molecule and the probe particle along the *z*-axis (perpendicular to the surface plane).

Supplementary Note 6. Chemical formula of β-cyclodextrin, showing the numbering and designation of atoms

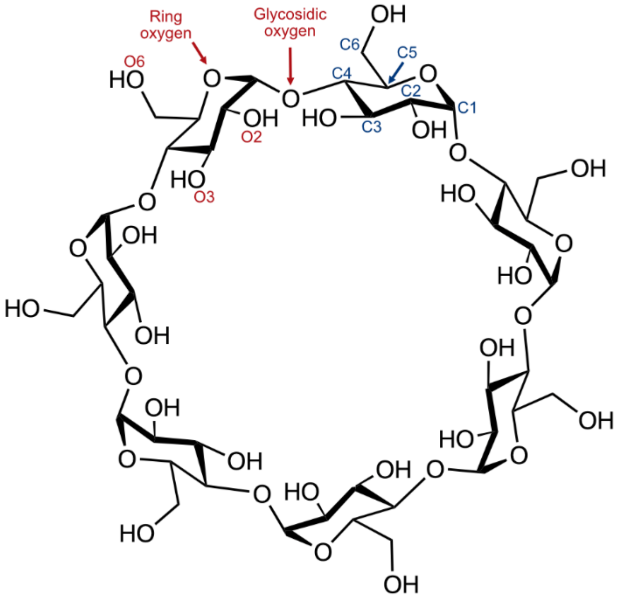

Supplementary Fig. 8 | Numbering and designation of carbon and oxygen atoms in β-CD. This nomenclature is employed throughout the present study. The same numbering applies to all seven glucose residues.

Supplementary Note 7. Additional STM and nc-AFM images of individual β-cyclodextrin molecules

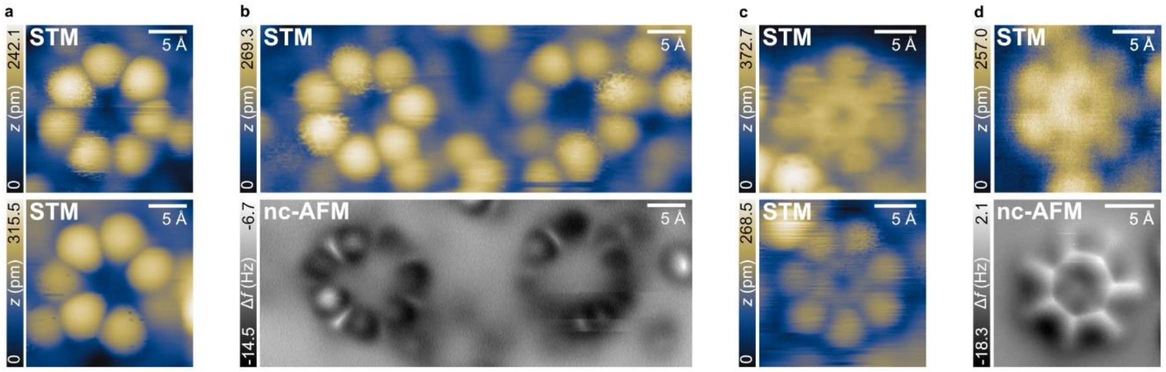

Supplementary Fig. 9 | Constant-current STM and constant-height nc-AFM images of individual β-CD molecules on Au(111). a, STM topographic images of two different β-CD molecules in the s↑ geometry. **b**, Corresponding STM and nc-AFM image of the same region showing two β-CD molecules in s↑ orientation. **c**, STM images of two different β-CD molecules in p↑ orientation. **d**, Corresponding STM and nc-AFM image of the same β-CD molecule in p↑ orientation.

Supplementary references

1. Schuler, B., Liu, W., Tkatchenko, A., Moll, N., Meyer, G., Mistry, A., Fox, D. & Gross, L. Adsorption Geometry Determination of Single Molecules by Atomic Force Microscopy. *Phys. Rev. Lett.* **111**, 106103 (2013).

2. Van Der Spoel, D., Lindahl, E., Hess, B., Groenhof, G., Mark, A. E. & Berendsen, H. J. C. GROMACS: Fast, flexible, and free. *J. Comput. Chem.* **26**, 1701-1718 (2005).

3. Tribello, G. A., Bonomi, M., Branduardi, D., Camilloni, C. & Bussi, G. PLUMED 2: New feathers for an old bird. *Comput. Phys. Commun.* **185**, 604-613 (2014).

4. Kirschner, K. N. & Woods, R. J. Solvent interactions determine carbohydrate conformation. *Proc. Natl. Acad. Sci.* **98**, 10541-10545 (2001).

5. Basma, M., Sundara, S., Çalgan, D., Vernali, T. & Woods, R. J. Solvated ensemble averaging in the calculation of partial atomic charges. *J. Comput. Chem.* **22**, 1125-1137 (2001).

6. Kirschner, K. N. & Woods, R. J. Quantum Mechanical Study of the Nonbonded Forces in Water–Methanol Complexes. *J. Phys. Chem. A* **105**, 4150-4155 (2001).

7. Cézard, C., Trivelli, X., Aubry, F., Djedaïni-Pilard, F. & Dupradeau, F.-Y. Molecular dynamics studies of native and substituted cyclodextrins in different media: 1. Charge derivation and force field performances. *Phys. Chem. Chem. Phys.* **13**, 15103-15121 (2011).

8. Iori, F., Di Felice, R., Molinari, E. & Corni, S. GolP: An atomistic force-field to describe the interaction of proteins with Au(111) surfaces in water. *J. Comput. Chem.* **30**, 1465-1476 (2009).

9. Kührová, P., Best, R. B., Bottaro, S., Bussi, G., Šponer, J., Otyepka, M. & Banáš, P. Computer Folding of RNA Tetraloops: Identification of Key Force Field Deficiencies. *J. Chem. Theory Comput.* **12**, 4534-4548 (2016).

10. Hess, B., Bekker, H., Berendsen, H. J. C. & Fraaije, J. G. E. M. LINCS: A linear constraint solver for molecular simulations. *J. Comput. Chem.* **18**, 1463-1472 (1997).

11. Bussi, G., Donadio, D. & Parrinello, M. Canonical sampling through velocity rescaling. *J. Chem. Phys.* **126**, 014101 (2007).

12. Berendsen, H. J. C., Postma, J. P. M., van Gunsteren, W. F., DiNola, A. & Haak, J. R. Molecular dynamics with coupling to an external bath. *J. Chem. Phys.* **81**, 3684-3690 (1984).

13. Blum, V., Gehrke, R., Hanke, F., Havu, P., Havu, V., Ren, X., Reuter, K. & Scheffler, M. Ab initio molecular simulations with numeric atom-centered orbitals. *Comput. Phys. Commun.* **180**, 2175-2196 (2009).

14. Perdew, J. P., Burke, K. & Ernzerhof, M. Generalized Gradient Approximation Made Simple. *Phys. Rev. Lett.* **77**, 3865-3868 (1996).

15. Hapala, P., Kichin, G., Wagner, C., Tautz, F. S., Temirov, R. & Jelínek, P. Mechanism of high-resolution STM/AFM imaging with functionalized tips. *Phys. Rev. B* **90**, 085421 (2014).
