# Supplementary material for: Atomically resolved imaging of the conformations and adsorption geometries of individual β-cyclodextrins with non-contact AFM
Source: Nat Commun. 2024 Nov 2;15:9482. doi: 10.1038/s41467-024-53555-0 (PMC11531514; doi:10.1038/s41467-024-53555-0)
Supplement: Supplementary file 2 — Description of Additional Supplementary Files [file 41467_2024_53555_MOESM2_ESM.pdf]

Description of Additional Supplementary Files

File Name: Supplementary Data 1

Description: DFT-optimized seconday face-up structure of cyclodextrin in XYZ format.

File Name: Supplementary Data 2

Description: DFT-optimized seconday face-up structure of cyclodextrin in XYZ format.
